# Is the use of cholesterol in mortality risk algorithms in clinical guidelines valid? Ten years prospective data from the Norwegian HUNT 2 study

**DOI:** 10.1111/j.1365-2753.2011.01767.x

**Published:** 2012-02

**Authors:** Halfdan Petursson, Johann A Sigurdsson, Calle Bengtsson, Tom I L Nilsen, Linn Getz

**Affiliations:** 1 Research Fellow, Research Unit of General Practice, Department of Public Health and General Practice, Norwegian University of Science and Technology (NTNU)Trondheim, Norway; 2 Professor, Department of Family Medicine, University of Iceland, and Centre of Development, Primary Health Care of the Capital AreaReykjavik, Iceland; 3 Professor Emeritus, Department of Primary Health Care, The Sahlgrenska Academy, University of GothenburgGothenburg, Sweden; 4 Associate Professor, Department of Human Movement Science, Norwegian University of Science and Technology (NTNU)Trondheim, Norway; 5 Associate Professor, Research Unit of General Practice, Department of Public Health and General Practice, Norwegian University of Science and Technology (NTNU)Trondheim, Norway and Landspitali University Hospital, Reykjavik, Iceland

**Keywords:** cardiovascular risk estimation, cholesterol, clinical guidelines, preventive medicine, primary care, mortality

## Abstract

**Rationale, aims and objectives:**

Many clinical guidelines for cardiovascular disease (CVD) prevention contain risk estimation charts/calculators. These have shown a tendency to overestimate risk, which indicates that there might be theoretical flaws in the algorithms. Total cholesterol is a frequently used variable in the risk estimates. Some studies indicate that the predictive properties of cholesterol might not be as straightforward as widely assumed. Our aim was to document the strength and validity of total cholesterol as a risk factor for mortality in a well-defined, general Norwegian population without known CVD at baseline.

**Methods:**

We assessed the association of total serum cholesterol with total mortality, as well as mortality from CVD and ischaemic heart disease (IHD), using Cox proportional hazard models. The study population comprises 52 087 Norwegians, aged 20–74, who participated in the Nord-Trøndelag Health Study (HUNT 2, 1995–1997) and were followed-up on cause-specific mortality for 10 years (510 297 person-years in total).

**Results:**

Among women, cholesterol had an inverse association with all-cause mortality [hazard ratio (HR): 0.94; 95% confidence interval (CI): 0.89–0.99 per 1.0 mmol L^−1^ increase] as well as CVD mortality (HR: 0.97; 95% CI: 0.88–1.07). The association with IHD mortality (HR: 1.07; 95% CI: 0.92–1.24) was not linear but seemed to follow a ‘U-shaped’ curve, with the highest mortality <5.0 and ≥7.0 mmol L^−1^. Among men, the association of cholesterol with mortality from CVD (HR: 1.06; 95% CI: 0.98–1.15) and in total (HR: 0.98; 95% CI: 0.93–1.03) followed a ‘U-shaped’ pattern.

**Conclusion:**

Our study provides an updated epidemiological indication of possible errors in the CVD risk algorithms of many clinical guidelines. If our findings are generalizable, clinical and public health recommendations regarding the ‘dangers’ of cholesterol should be revised. This is especially true for women, for whom moderately elevated cholesterol (by current standards) may prove to be not only harmless but even beneficial.

## Introduction

It has long been considered ‘common knowledge’ that total serum cholesterol is an important and strong, independent risk factor for cardiovascular disease (CVD) [1–4]. This association has been deemed to be linear, meaning ‘the lower the total cholesterol level, the better’. During the last decades, CVD prevention has been marked by a trend of gradually lowering thresholds of risk definitions regarding cholesterol levels [2,5–8], in parallel with other CVD risk factors such as hypertension and blood sugar [5,7,9–12]. Campaigns aimed at the general public have underlined the risks associated with total cholesterol above 5.0 mmol L^−1^ (see, for instance, the 2011 Norwegian campaign ‘Under 5’ at http://www.under5.no).

In recent years, a ‘combined estimate’ [2,7,8,13–17] has become the most widespread method for CVD risk evaluation, as it is believed to have better predictive properties than the single risk factor approach. Most combined estimates include the risk factors of age, sex, smoking, blood pressure and serum cholesterol. Additionally, the algorithms include, to a varying degree, other factors such as diabetes, obesity, family history and cholesterol subfractions [low-density lipoprotein (LDL) and high-density lipoprotein (HDL) cholesterol] [2,5,7,8,12–17].

Some studies [18–22], including papers from our research group [23,24], have problematized overestimation of CVD risk in authoritative, preventive clinical guidelines. Both single risk factors [24,25] and combined risk estimates have been addressed [23,26]. We have shown that according to authoritative CVD guidelines, 75% of the adult Norwegian population would be deemed at risk for CVD and in need of clinical attention (advice and supervision) [25,26]. Consequently, we have questioned the theoretical basis of the guidelines. In the present study, we look specifically at the validity of guidelines' risk estimations involving cholesterol. In particular, we challenge the widespread assumption of a linear relationship between total cholesterol levels and disease development (expressed as mortality in our analysis).

We are well aware of evidence indicating that cholesterol subparticles, including various lipoproteins, may have stronger associations with CVD development than total cholesterol [27–34]. However, the emphasis on total cholesterol (as both a single risk factor and an element in multiple risk estimates) still prevails in many authoritative, clinical guidelines [1,7,17]. For instance, both the 2003 and the 2007 European Guidelines on CVD prevention state that ‘in general, total plasma cholesterol should be below 5,’ [2,16] and the risk charts in the same guidelines include total cholesterol.

In the past decades, a number of studies have found a strong and graded association between serum cholesterol and mortality from ischaemic heart disease (IHD) [14,35–48]. Regarding total mortality, however, the association has not been clear. Some studies have found no association, and others have even suggested an inverse relationship [38–41,43,49–61]. Some studies have shown an inverse or a U-shaped association between cholesterol and death from causes other than CVD, such as cancer [52,56,62,63]. The phrase ‘U-shaped association’ (alternatively ‘J-shaped’) indicates that higher mortality (or incidences) can be observed both in individuals with low and high levels of cholesterol compared with individuals with levels in between. It is important to note, however, that the phrase ‘U-shaped’ does not necessarily indicate that both arms of the ‘U’ are equal in terms of mortality rates or the proportion of the population belonging to each arm.

Regarding the association between cholesterol and overall CVD mortality, some studies have found no association or a U-shaped or even an inverse association [37,58,61,64–67]. This has been explained by an association with cerebral strokes (primarily haemorrhagic strokes), as opposed to heart disease [34,46,67–72]. Interestingly, some studies have also found an inverse [55,67,73] or a U-shaped [61,74–78] association with IHD incidence and mortality, primarily among individuals, aged 60 years and older.

The incidence, prevalence and mortality from CVD have decreased substantially throughout the Western world in recent decades [79,80]. There are also significant time trend changes regarding various risk factors for CVD [81–83]. Changes have occurred, both regarding risk factors frequently included in combined risk estimates [83–85] and for factors such as societal structure [86,87], pollution [88], television viewing [89] and dietary habits [90,91]. These on-going changes are likely to alter the predictive value of risk algorithms based on observational data collected years or even decades ago (retrospective risk bias). As cholesterol has become an essential part of lay-people's basic understanding of their health, and the prevalence of slightly ‘elevated’ cholesterol levels is so high, we believe that it is important to re-examine old assumptions regarding cholesterol as a risk factor.

The aim of the present study was to document the strength and validity of total serum cholesterol as a risk factor for mortality, as defined by current CVD prevention guidelines. For this purpose, we used data from a well-defined, general Norwegian population without known CVD at baseline. We focused on deaths from cardiovascular disease, IHD and death from all causes (total mortality) within a follow-up period of 10 years.

## Methods

### Study population

All adults, aged 20 years or older and living in Nord-Trøndelag County in Norway in 1995–1997, were invited to participate in the second wave of the Nord-Trøndelag Health Study (HUNT 2). Overall, 74% of women (34 786) and 65% of men (30 575) chose to participate. The HUNT 2 population is ethnically homogeneous (dominated by individuals of Nordic origin) and has been considered fairly representative of the total Norwegian population with respect to demography, socio-economic factors, morbidity and mortality, including mortality from CVD [92]. The HUNT 2 study has been described in detail elsewhere (see http://www.ntnu.no/hunt/english) [92].

For the purpose of the present analysis, the following HUNT 2 participants were excluded: 6780 individuals aged 75 years or more at baseline (2815 men and 3965 women); 3430 individuals (2207 men and 1223 women) with established CVD at baseline (self-reported myocardial infarction, stroke or angina pectoris); and 3064 persons with missing data on one or more of the following variables: serum cholesterol, systolic blood pressure and smoking status. Our calculations are thereby based on information from 52 087 individuals (24 235 men and 27 852 women) aged 20–74 years and free from known CVD at baseline.

### Study variables

In the HUNT 2 survey, total serum cholesterol was measured by an enzymatic colorimetric cholesterol esterase method [92]. The blood pressure of persons in a seated position was measured by a specially trained personnel using Dinamap 845XT, based on oscillometry. The cuff size was adjusted after measuring the arm circumference, and blood pressure was recorded as the mean values of the second and third measurements performed consecutively at the same visit. Smoking was defined as daily smoking of cigarettes, cigars or a pipe.

### Follow-up

The personal identity number of Norwegian citizens enabled linking of HUNT 2 participant data to the Cause of Death Registry at Statistics Norway (information on http://www.ssb.no/english/). For the present analysis, each participant contributed person–time from the date of clinical examination (August 1995–June 1997) until 10 years of follow-up had been achieved (until August 2005–June 2007, depending on participation dates) or until the date of death if this occurred in the follow-up period, making the oldest participants of the study 84 years of age at the end of the follow-up. The follow-up time came to a total of 510 297 person-years. Death from CVD was defined by the International Classification of Disease code for the primary diagnosis of death (ICD-9: 390-459; ICD-10: I 00-I 99) as well as death from IHD (ICD-9: 410-414; ICD-10: I 20-I 25).

### Statistical analysis

The first part of our analysis involved making a simple CVD risk estimation chart to compare with the charts currently recommended for clinical practice in Norway. We used the Systematic Coronary Risk Evaluation (SCORE) chart of the European Society of Cardiology [2,15] and the nationally adjusted chart used in the Norwegian National Guidelines [17] as a reference. These charts are intended to depict the 10-year risk of dying from CVD, given the level of risk factors at baseline: sex, age, smoking status, systolic blood pressure and total cholesterol. To have a meaningful amount of data for each square of our chart, we based it on three age groups (20–39, 40–59 and 60–74 years), two levels of systolic blood pressure (<140 mm Hg vs. ≥140 mm Hg, in accordance with guidelines), smokers vs. non-smokers, and two levels of total cholesterol. Regarding cholesterol, the levels <5.5 mmol L^−1^ vs. ≥5.5 mmol L^−1^ were used (cut-off approximately 215 mg dL^−1^). This cut-off point assigns 40% of participating males and 43% of females to the ‘low level’ category. Using a cut-off point of 5.0 mmol L^−1^ (which guidelines [2,16] state that cholesterol should be below) would have assigned only 24% of males and 27% of females to the lower cholesterol stratum. The median cholesterol level of the participants was 5.7 mmol L^−1^ for both genders. The observed mortality rates per 1000 person-years were calculated for each square of the chart.

For the next part of our analysis, we used Cox proportional hazard models to compute hazard ratios (HRs) for overall mortality and mortality from CVD and IHD, associated with different levels of cholesterol at baseline. The precision of the estimated associations was assessed by a 95% confidence interval (CI). Departure from the proportional hazard assumptions was evaluated by Schoenfeld residuals.

We computed sex-specific HRs for cholesterol as a continuous variable as well as a variable with four categories (<5.0, 5–5.9, 6.0–6.9 and ≥7.0 mmol L^−1^). We adjusted for the other variables of the aforementioned chart, namely age (in the timescale), systolic blood pressure (as a continuous variable) and smoking status. We also ran an alternative model including the same variables, in addition to waist-to-hip ratio (WHR), level of physical activity, self-reported diabetes mellitus and family history of CVD. The categorical cholesterol variable was tested for linear as well as quadratic trend. Finally, we conducted an analysis of cholesterol as a dichotomous variable with the cut-off point of 5.5 mmol L^−1^, stratified by smoking status, and an analysis of the effect of smoking stratified by the dichotomous cholesterol variable for comparison.

All statistical tests were two-sided and all analyses were performed using Stata for Windows (version 11; StataCorp LP, TX, USA).

### Ethics statement

Each participant in the HUNT study signed a written consent regarding the screening and the use of data for research purposes as well as linking their data to other registers (subject to the approval of the Norwegian Data Inspectorate). The study was approved by the Norwegian Data Inspectorate and the Regional Committee for Ethics in Medical Research.

## Results

**Figure 1:**
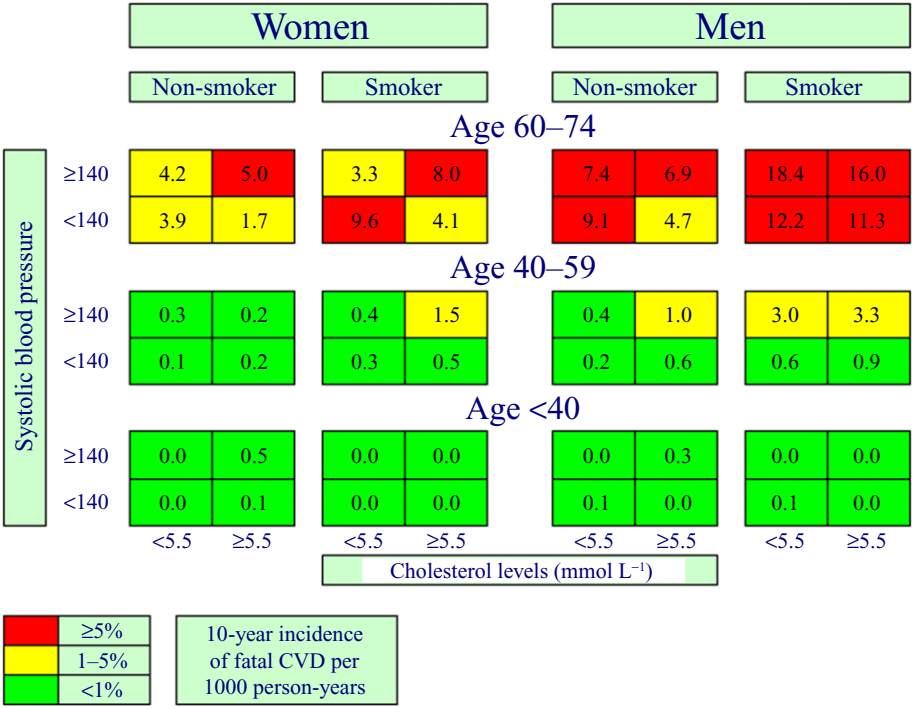
Ten-year incidence of fatal cardiovascular disease per 1000 person-years in the population of Nord-Trøndelag (HUNT 2 study).

Figure 1 shows CVD mortality for the HUNT 2 population during the 10-year follow-up period (mortality rates per 1000 person-years), according to each level of the risk factors found in the international SCORE system. This model showed a general trend towards increased mortality for an increase in any of the included risk factors, except for cholesterol, where no such association was observed. The results were similar regarding all-cause mortality and IHD mortality (data not shown).

Table 1 shows the sex-specific associations of different levels of serum cholesterol with mortality, both total mortality and CVD and IHD mortality. Among women, serum cholesterol had an inverse association with all-cause mortality as well as CVD mortality (although not reaching statistical significance) (Table 1). The association with IHD mortality appeared to follow a U-shaped curve. Test for quadratic trend did not support the existence of a U-shaped curve (*P* = 0.16).

**Table 1:** Risk of death from all causes, cardiovascular disease and ischaemic heart disease among individuals aged 20–74; associations of total cholesterol with mortality

		All causes			Cardiovascular disease			Ischaemic heart disease		
				
Cholesterol (mmol L^−1^)	No. of persons	No. of deaths	Adjusted* HR (95% CI)	*P*_trend_	No. of deaths	Adjusted* HR (95% CI)	*P*_trend_	No. of deaths	Adjusted* HR (95% CI)	*P*_trend_
**Men**										
<5.0	5 918	208	1.00 (Reference)		58	1.00 (Reference)		24	1.00 (Reference)	
5.0–5.9	8 021	410	0.77 (0.65–0.92)		132	0.80 (0.59–1.09)		65	0.94 (0.59–1.50)	
6.0–6.9	6 658	500	0.84 (0.71–0.99)		168	0.87 (0.64–1.18)		85	1.06 (0.67–1.67)	
≥7.0	3 638	329	0.89 (0.74–1.06)	0.90	128	1.05 (0.76–1.44)	0.25	57	1.12 (0.69–1.81)	0.39
per unit increase	24 235	1447	0.98 (0.93–1.03)	0.35	486	1.06 (0.98–1.15)	0.17	231	1.08 (0.96–1.22)	0.18
**Women**										
<5.0	7 613	98	1.00 (Reference)		19	1.00 (Reference)		9	1.00 (Reference)	
5.0–5.9	8 565	243	0.92 (0.72–1.17)		59	0.90 (0.53–1.52)		19	0.61 (0.27–1.38)	
6.0–6.9	6 404	327	0.84 (0.66–1.06)		91	0.81 (0.49–1.35)		32	0.60 (0.28–1.30)	
≥7.0	5 270	375	0.72 (0.57–0.92)	0.001	121	0.74 (0.44–1.22)	0.13	56	0.72 (0.34–1.51)	1.00
per unit increase	27 852	1043	0.94 (0.89–0.99)	0.02	290	0.97 (0.88–1.07)	0.53	116	1.07 (0.92–1.24)	0.37

* Adjusted for age (in the timescale), smoking (current vs. not) and systolic blood pressure (continuous).

CI, confidence interval; HR, hazard ratio.

**Figure 2:**
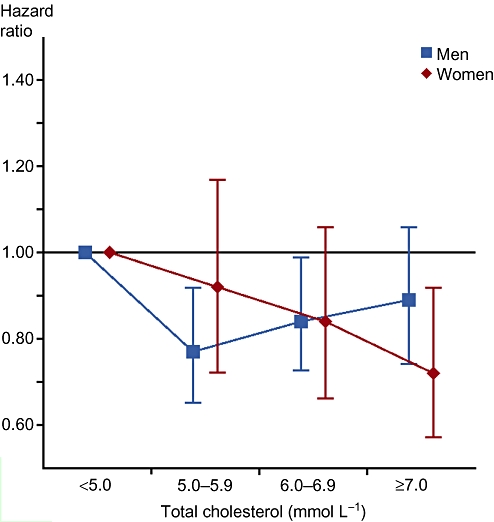
Risk of death (all causes) associated with different levels of total cholesterol. Hazard ratios and 95% confidence intervals for men (blue box) and women (red diamond) separately. Adjusted for age, smoking and systolic blood pressure.

**Figure 3:**
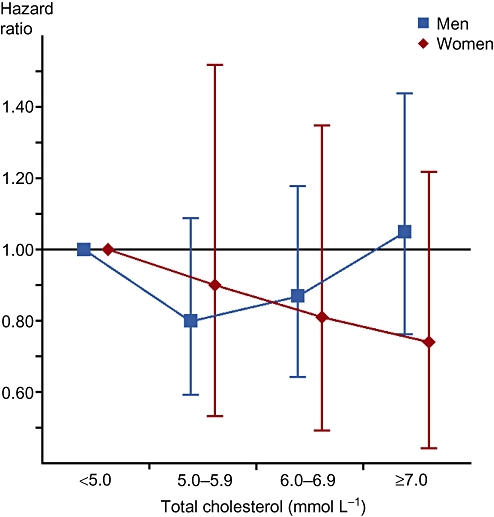
Risk of death from cardiovascular disease associated with different levels of total cholesterol. Hazard ratios and 95% confidence intervals for men (blue box) and women (red diamond). Adjusted for age, smoking and systolic blood pressure.

**Figure 4:**
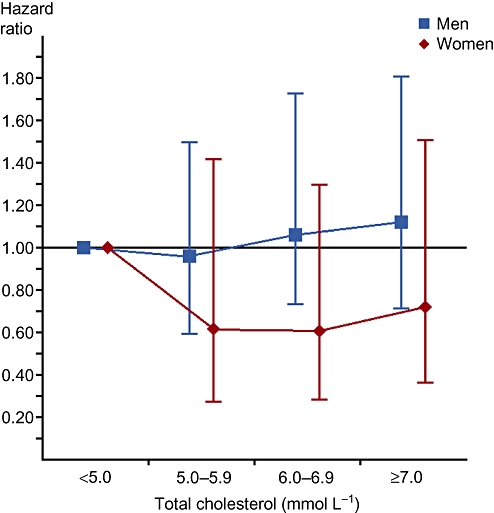
Risk of death from ischaemic heart disease associated with different levels of total cholesterol. Hazard ratios and 95% confidence intervals for men (blue box) and women (red diamond). Adjusted for age, smoking and systolic blood pressure.

Among men, cholesterol did not seem to be linearly associated with mortality but rather the association followed a U-shaped pattern, with the lowest mortality appearing in the second cholesterol category (5.0–5.9 mmol L^−1^). This was apparent in all mortality categories. Consequently, cholesterol analysed as a continuous variable did not show a statistically significant linear association with mortality. Test for quadratic trend yielded *P* = 0.01 for all-cause mortality (indicating a true U-curve), *P* = 0.055 for CVD (approaching statistical significance) and *P* = 0.80 for IHD (practically excluding a U-curve). The associations between cholesterol and mortality are visualized in Figs 2–4.

A sensitivity analysis adjusting for four additional risk factors (WHR, physical activity and family history) revealed results of no considerable difference from the first model (adjusting for age, smoking and systolic blood pressure) for either sex.

The association of a dichotomous cholesterol variable with mortality, stratified by smoking status, is shown in Table 2. The HRs relate to the risk of dying among individuals with high serum cholesterol (≥5.5 mmol L^−1^) compared with those with lower levels (<5.5 mmol L^−1^). Having cholesterol levels above 5.5 mmol L^−1^ was not associated with increased mortality, either among smokers or among non-smokers.

**Table 2:** Risk of death from all causes, cardiovascular disease and ischaemic heart disease depending on smoking status for individuals 20–74 years old; hazard ratios for high* total cholesterol compared with low cholesterol levels

		Men			Women		
			
Cause of death	Level of cholesterol*	No. of persons	No. of deaths	Adjusted† HR (95% CI)	No. of persons	No. of deaths	Adjusted† HR (95% CI)
**All causes**							
Smokers	High	4726	476	0.87 (0.73–1.03)	5 406	339	1.00 (0.77–1.31)
	Low	2680	180	1.00 (Reference)	3 795	75	1.00 (Reference)
Non-smokers	High	9724	573	0.94 (0.80–1.10)	10 485	503	0.74 (0.60–0.91)
	Low	7105	218	1.00 (Reference)	8 166	126	1.00 (Reference)
**CVD**							
Smokers	High	4726	181	0.92 (0.68–1.24)	5 406	94	1.09 (0.60–2.00)
	Low	2680	58	1.00 (Reference)	3 795	13	1.00 (Reference)
Non-smokers	High	9724	182	0.91 (0.68–1.21)	10 485	160	1.00 (0.63–1.57)
	Low	7105	65	1.00 (Reference)	8 166	23	1.00 (Reference)
**IHD**							
Smokers	High	4726	89	1.03 (0.66–1.59)	5 406	46	1.19 (0.48–2.93)
	Low	2680	26	1.00 (Reference)	3 795	6	1.00 (Reference)
Non-smokers	High	9724	88	1.00 (0.65–1.53)	10 485	56	0.96 (0.45–2.06)
	Low	7105	28	1.00 (Reference)	8 166	8	1.00 (Reference)

* Comparison of high (≥5.5 mmol L^−1^) vs. low (<5.5 mmol L^−1^) total cholesterol.

† Adjusted for age (in the timescale), smoking (current vs. not) and systolic blood pressure.

CVD, cardiovascular disease; CI, confidence interval; HR, hazard ratio; IHD, ischaemic heart disease.

Smoking, on the other hand, was strongly associated with increased mortality in all mortality categories among both sexes (Table 3). Among women, the association was somewhat stronger for those with cholesterol below 5.5 mmol L^−1^.

**Table 3:** Risk of death from all causes, cardiovascular disease and ischaemic heart disease depending on levels of total cholesterol for individuals 20–74 years old; hazard ratios for smoking compared with non-smoking

		Men			Women		
			
Cause of death	Smoking status	No. of persons	No. of deaths	Adjusted* HR (95% CI)	No. of persons	No. of deaths	Adjusted* HR (95% CI)
**All causes**							
High† cholesterol	Smoking	4726	476	2.09 (1.71–2.55)	5 406	339	1.72 (1.29–2.30)
	Non-smoking	2680	180	1.00 (Reference)	3 795	75	1.00 (Reference)
Low‡ cholesterol	Smoking	9724	573	1.99 (1.76–2.24)	10 485	503	2.16 (1.87–2.48)
	Non-smoking	7105	218	1.00 (Reference)	8 166	126	1.00 (Reference)
**CVD**							
High cholesterol	Smoking	4726	181	2.30 (1.61–3.29)	5 406	94	1.82 (0.91–3.64)
	Non-smoking	2680	58	1.00 (Reference)	3 795	13	1.00 (Reference)
Low cholesterol	Smoking	9724	182	2.44 (1.98–3.00)	10 485	160	2.24 (1.73–2.91)
	Non-smoking	7105	65	1.00 (Reference)	8 166	23	1.00 (Reference)
**IHD**							
High cholesterol	Smoking	4726	89	2.28 (1.34–3.90)	5 406	46	2.35 (0.80–6.95)
	Non-smoking	2680	26	1.00 (Reference)	3 795	6	1.00 (Reference)
Low cholesterol	Smoking	9724	88	2.42 (1.80–3.25)	10 485	56	3.08 (2.07–4.58)
	Non-smoking	7105	28	1.00 (Reference)	8 166	8	1.00 (Reference)

* Adjusted for age (in the timescale), smoking (current vs. not) and systolic blood pressure.

† High total cholesterol ≥5.5 mmol L^−1^.

‡ Low total cholesterol <5.5 mmol L^−1^.

CVD, cardiovascular disease; CI, confidence interval; HR, hazard ratio; IHD, ischaemic heart disease.

## Discussion

In this validation study of current guidelines for CVD prevention, which is based on new epidemiological data from a large and representative Norwegian population, we found total cholesterol to be an overestimated risk factor.

Regarding the association between total cholesterol and mortality, our results generally indicated U-shaped or inverse linear curves for total and CVD mortality. Only the association with IHD among men could be interpreted as suggesting a positive, linear trend.

Our results contradict the guidelines' well-established demarcation line (5 mmol L^−1^) between ‘good’ and ‘too high’ levels of cholesterol. They also contradict the popularized idea of a positive, linear relationship between cholesterol and fatal disease. Guideline-based advice regarding CVD prevention may thus be outdated and misleading, particularly regarding many women who have cholesterol levels in the range of 5–7 mmol L^−1^ and are currently encouraged to take better care of their health.

Our finding of significant discrepancies between epidemiological data and clinical guidelines [2,16,17], suggesting a linear relation between total cholesterol and mortality from CVD is in accord with other studies [61,66,67].

The main strengths of our study are the prospective and comprehensive nature of the HUNT 2 survey, its good participation rates, and representativeness of the entire Norwegian population of similar, i.e. Nordic, origin. A corresponding weakness is the lack of immediate generalizability to Norwegians with other ethnic backgrounds. Another potential weakness of our study is the lack of information about cholesterol-lowering drug treatment among the participants. However, this is unlikely to be an important source of bias as our population was free from CVD at baseline, and cholesterol-lowering drugs were not recommended for primary prevention in the study period.

It is possible that the Norwegian HUNT 2 population differs somewhat from earlier study populations in levels of CVD risk factors and mortality, and that this may affect (or confound) the association of cholesterol with mortality. Norway is an affluent country, and Norwegians are currently one of the longest lived people in the world [93]. The rate of smoking among men is relatively low, by international comparison [93]. The stable social structure could also play a part, including a well-functioning health care system with good access and coverage for all.

Various studies have shown cholesterol, smoking and high blood pressure to have a multiplicative effect on IHD risk rather than an additive effect [94–97]. It may be that cholesterol acts differently as a risk factor for IHD than previously believed, at least in certain risk factor combinations and/or under certain developmental and contextual circumstances, such as those mentioned earlier. At least in some settings, cholesterol may represent a risk marker and/or a weak risk factor rather than an important one. More valid risk factors might be found by further investigation of lipoproteins and/or other subparticles of cholesterol, but the same dilemmas may arise in relation to those entities. What appears evident, however, is that more updated and complex disease prediction models are needed.

Regarding the immediate future of guidelines and combined risk estimates for CVD, we envisage three options: first, IHD mortality (and not overall CVD mortality) might be considered an appropriate end point for the current risk estimates. However, our results (Table 1) indicate that even such a limited focus would be problematic, at least for women. Alternatively, total cholesterol could be excluded from the risk estimates, potentially being replaced either by nothing or by some different subparticle(s) of cholesterol with better predictive properties, such as HDL or HDL/total cholesterol [2,8,16]. Finally, future risk estimates may be based on more nuanced statistical models, allowing for gender- and age-specific associations between cholesterol and disease development (mortality).

The Norwegian guidelines for prevention of CVD [17] include a risk estimation model developed on the basis of Norwegian population data [21]. This model assumes a linear association of cholesterol with CVD mortality, and the authors do not indicate that any evaluation of the linearity of the association has taken place, i.e. an evaluation of a possible U-shaped association. Selmer *et al*. included participants, aged 20–67, in their study, while we included people up to 74 years old, and the U-shaped association has been most prominent in studies, including participants over age 60. This difference should, however, be minimized by the statistical adjustments made for the effects of age. Besides the included age groups, there is no reason to believe that the Norwegian population data underlying the Norwegian guidelines differ considerably from the HUNT 2 population.

To address the question of whether the U-shaped association was age-dependent in the HUNT population, we performed an age-stratified Cox-regression analysis post-hoc. The results indicated a U-shaped association of cholesterol with CVD mortality among men aged 40–74, and an inverse association among women aged 60–74. Because of limited statistical power, we refrain from emphasizing these results. Seen in the light of previous studies [37,55,58,61,64–66,73–77], it is possible that a U-shaped association is primarily a phenomenon related to people aged 60 years and older.

Our smoking-stratified analysis (Table 2) indicated that the U-shaped association can possibly be found among both smokers and non-smokers, as no considerable difference was observed between these two strata. Analysis with more categories of cholesterol levels would have been preferable here, but due to limited statistical power, we refrain from further analyses post-hoc.

In contrast to cholesterol, the detrimental effect of smoking was clearly evident even after stratifying for cholesterol levels (Table 3). This emphasizes the importance of smoking as a CVD risk factor compared with cholesterol.

## Conclusions

Based on epidemiological analysis of updated and comprehensive population data, we found that the underlying assumptions regarding cholesterol in clinical guidelines for CVD prevention might be flawed: cholesterol emerged as an overestimated risk factor in our study, indicating that guideline information might be misleading, particularly for women with ‘moderately elevated’ cholesterol levels in the range of 5–7 mmol L^−1^. Our findings are in good accord with some previous studies. A potential explanation of the lack of accord between clinical guidelines and recent population data, including ours, is time trend changes for CVD/IHD and underlying causal (risk) factors.

‘Know your numbers’ (a concept pertaining to medical risk factor levels, including cholesterol) is currently considered part of responsible citizenship, as well as an essential element of preventive medical care. Many individuals who could otherwise call themselves healthy struggle conscientiously to push their cholesterol under the presumed ‘danger’ limit (i.e. the recommended cut-off point of 5 mmol L^−1^), coached by health personnel, personal trainers and caring family members. Massive commercial interests are linked to drugs and other remedies marketed for this purpose. It is therefore of immediate and wide interest to find out whether our results are generalizable to other populations.

## Funding

This work was supported by the Research Unit of General Practice, Department of Public Health and General Practice, Norwegian University of Science and Technology (NTNU), Trondheim, Norway; the Norwegian Medical Association's Funds for Research in General Practice; and the Research Fund of the Icelandic College of Family Physicians.
